# Professor Barrett's Paper before the Institute of Heating and Ventilating Engineers

**Published:** 1905-07-29

**Authors:** W. F. Barrett

**Affiliations:** Dublin


### EDITOR'S LETTER-BOX.

### PROFESSOR BARRETT'S PAPER BEFORE THE INSTITUTE OF HEATING AND VENTILATING ENGINEERS.

SIR,—It is hardly necessary to reply to your anonymous contributor. Any tyro in chemistry would tell him that when a gas flame is burning in an ordinary glass globe open above and below the combustion is more perfect than when the upper opening is closed, the lower remaining as before. Substituting an iron for a glass globe makes no difference in this result, however much the products of combustion are heated, and the only reason why a gas flame enclosed in such an iron globe warms a small room more effectually than a similar gas flame in the usual glass globe is, as I incidentally remarked in my address, because of the greater radiation which takes place from the heated iron globe.

Yours obediently,

Dublin, July 22. W. F. BARRETT.

